# Bovine brucellosis trends in Malaysia between 2000 and 2008

**DOI:** 10.1186/1746-6148-9-230

**Published:** 2013-11-18

**Authors:** Mukhtar S Anka, Latiffah Hassan, Azri Adzhar, Siti Khairani-Bejo, Ramlan Bin Mohamad, Mohamed A Zainal

**Affiliations:** 1 Department of Veterinary Pathology and Microbiology, Faculty of Veterinary Medicine, Universiti Putra Malaysia, UPM, Serdang 43400, Malaysia; 2 Epidemiology and Surveillance Unit, Department of Veterinary Services, Putrajaya, Malaysia; 3 Veterinary Research Institute, 59 Jalan Sultan Azlan Shah, Ipoh, Perak 31400, Malaysia; 4 Department of Agribusiness and Information System, Faculty of Agriculture, Universiti Putra Malaysia, UPM, Serdang 43400, Malaysia

**Keywords:** Epidemiology, Bovine brucellosis, Distribution, Trend, Pattern

## Abstract

**Background:**

Bovine brucellosis is an important disease affecting cattle characterised by abortion, still birth, reduced milk production, weak foetus and infertility in both males and females. There is wide distribution of the disease among cattle and several wildlife species. Bovine brucellosis is commonly caused by *B. abortus* and very occasionally *B. melitensis* and *B. suis.* The distribution of bovine brucellosis in cattle has not been described in Malaysia. In this paper we describe the distribution, pattern and trend of bovine brucellosis in Peninsular Malaysia between 2000 and 2008 based on serological data obtained from nationwide *B. abortus* serosurveillance activities in cattle populations.

**Results:**

*Brucella* antibodies were detected in 21.8% of sampled herds (95% CI, 21.01–22.59) and 2.5% (95% CI; 2.45–2.55) of sampled cattle. The state of Pahang had the highest animal and herd-level seroprevalence of 5.3 and 43.6%, respectively. The herd-level seroprevalence varied but remained high (18-26%) over the period of study and generally increased from 2000 to 2008. Seropositive herds clustered around the central part of the peninsula within the period of the study. The months of September, October and November illustrated the highest rates with corresponding seroprevalences of 33.2, 38.4 and 33.9%, respectively. A noticeable variation was observed in the cattle-level seroprevalence, but the rate remained relatively low (<5%). The chi-square statistics showed herd size (χ2 = 1206.077, df = 2, p = 0.001), breed (χ2 = 37.429, df = 1, p = 0.001), month of sampling (χ2 = 51.596, df = 11 p = 0.001), year (χ2 = 40.08, df = 8, p = 0.001) and state (χ2 = 541.038, df = 10, p = 0.001) to be associated with increased seropositivity.

**Conclusion:**

Bovine brucellosis is widespread among herds in Peninsular Malaysia at a low within-herd seroprevalence rate.

## Background

Bovine brucellosis continues to be a common zoonosis disease with a significant economic impact in livestock that is widely distributed among cattle and related wildlife species worldwide [1-3]. The disease is primarily caused by *B. abortus* and occasionally *B. melitensis* and *B. suis*. Most human brucellosis cases, however, have been linked to *B. melitensis*[4]. Bovine brucellosis is characterised by abortion, still birth, infertility and reproductive failure [5]. Humans may contract the infection via direct contact of contaminated materials or drinking raw milk from affected cows [6]. In recent years, several outbreaks of brucellosis have been reported among humans in Malaysia, mainly due to the consumption of raw goat’s milk contaminated with *B. melitensis*[7,8]. Elsewhere, many brucellosis cases in humans have been attributed to drinking raw cow’s milk [9,10].

Bovine brucellosis was first identified in Malaysia in 1950 [11]. The spread of the disease later instigated a nationwide brucellosis eradication programme, which involved the testing and slaughter of seropositive animals and consequently resulted in a marked decline in the number of seropositive cattle [12]. Much success has been achieved through this programme and, consequently, resulted in a marked reduction in the number of seropositive cattle from 8.7% in 1980 to 0.4% in 1993 [13].

In many countries, serological testing followed by culling has been practiced to control brucellosis with varying levels of success [14]. In an effort committed to tackling the problem, the Malaysian veterinary authorities have conducted an active serosurveillance of bovine brucellosis for many years. The exercise is routinely followed by culling of infected animals with compensation to the farmers [12]. Previous surveys have established that bovine brucellosis may be hypoendemic but still occurs in many parts of the peninsula [15-18]. However, in the last decade, anecdotal evidence suggests an increase of brucellosis infection among cattle. In this study, we describe the trends and pattern of brucellosis among cattle in the past decade from a retrospective analysis of data collected from a nationwide brucellosis active surveillance programme. We believe that information from this study will enhance understanding about the epidemiology of bovine brucellosis in Peninsular Malaysia and assist the authorities in improving their disease-control strategies.

## Results

**Figure 1:**
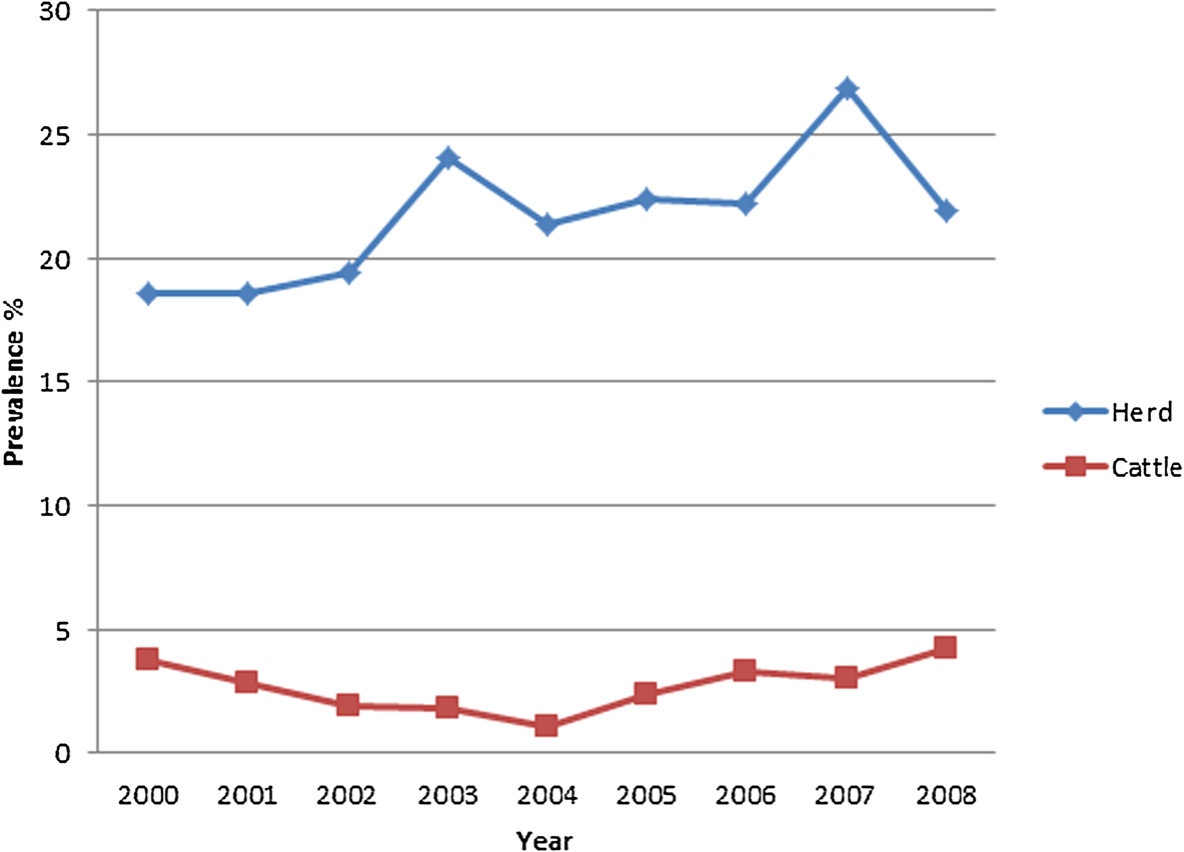
Annual herd-level and cattle seroprevalence of bovine brucellosis in Peninsular Malaysia from 2000 to 2008.

A total of 10,584 herds and 407,646 cattle were sampled within the period of study (2000–2008), of which 2,302 (21.8%; 95% CI, 21.01–22.59) herds and 10,013 (2.5%; 95% CI; 2.45–2.55) cattle tested positive. The annual mean seroprevalence level among cattle for the period of study was 2.7%, with 2008 having the highest rate (4.2% CI, 3.96–4.44) and 2004 having the lowest (1.1% CI, 1.03–1.17) (Figure 1). A significant decreasing trend of seroprevalence from 2000 to 2004 and increasing trend from 2004 to 2008 was observed (χ2 = 40.08, df = 8, *p* = 0.001) (Figure 1). Among the states, the highest cattle-level seroprevalence was observed in Pahang at 5.4% (95% CI, 4.76–5.84) and the lowest in Pulau Pinang at 1.2% (95% CI, 1.05–1.55) (Table 1).

**Table 1:** Herd-level seroprevalence of bovine brucellosis based on several factors in Peninsular Malaysia between 2000 and 2008

**Variables**	**Categories**	**N**	**Prevalence (%)**	**OR**	**95% CI**	**P-value**
Year	2000	1245	18.6	Ref	-	-
	2001	1227	18.6	1.00	0.818–1.227	0.986
	2002	1161	19.9	1.09	0.890–1.336	0.404
	2003	1427	20.4	1.12	0.928–1.363	0.232
	2004	1140	21.5	1.20	0.983–1.469	0.073
	2005	934	22.4	1.27	1.026–1.561	0.028
	2006	1289	22.1	1.25	1.026–1.513	0.026
	2007	1249	26.8	1.61	1.331–1.945	0.001
	2008	923	23.8	1.37	1.116–1.692	0.003
Month of sampling	January	476	17.2	Ref	-	-
	February	729	19.2	1.1	0.839–1.532	0.415
	March	1116	19.7	1.2	0.883–1.549	0.270
	April	1264	18.8	1.1	0.839–1.459	0.475
	May	1260	18.3	1.1	0.811–1.413	0.631
	June	1241	22.2	1.4	1.038–1.792	0.026
	July	1057	23.9	1.5	1.138–1.979	0.004
	August	1106	21.5	1.3	0.990–1.726	0.059
	September	762	24.9	1.6	1.186–2.115	0.002
	October	667	27.7	1.8	1.366–2.451	0.001
	November	502	25.3	1.6	1.182–2.206	0.003
	December	418	22.7	1.4	1.008–1.951	0.045
States	Selangor	1553	20.9	Ref	-	-
	Terengganu	545	18.5	0.9	0.671–1.102	0.232
	Perlis	52	36.5	2.2	1.221–3.876	0.008
	Perak	1935	19.2	0.9	0.761–1.062	0.212
	Pahang	1318	43.6	2.9	2.475–3.454	0.001
	Pulau Pinang	109	11.9	0.5	0.283–0.925	0.027
	N. Sembilan	1171	14.4	0.6	0.520–0.781	0.001
	Melaka	1016	10.8	0.5	0.364–0.579	0.001
	Kelantan	663	28.1	1.5	1.196–1.816	0.001
	Kedah	626	15.3	0.7	0.533–0.878	0.003
	Johor	1607	19.3	0.9	0.759–1.075	0.251
Herd size	<20	4328	9.0	Ref	-	-
	20–40	2101	27.5	3.1	2.660–3.510	0.001
	>40	1891	69.2	7.7	6.794–8.731	0.001
Breed	Kedah-Kelantan	3291	27.7	Ref	-	-
	Bali	25	36	1.5	0.648–3.342	0.356
	Brahman	283	38.9	1.7	1.294–2.138	0.001
	Kedah-Kelantan cross	3290	27.6	0.6	0.526–0.658	0.001
	Local Indian Dairy	3849	18.4	0.7	0.599–0.818	0.001
	Nellore	30	36.7	1.5	0.718–3.196	0.276
	Sahiwal-Friesien	1078	12.9	0.4	0.319–0.470	0.001
	Others	69	4.4	0.1	0.037–0.379	0.001
Type of production	Dairy	2333	17.2	Ref	-	-
	Beef	7538	23.2	1.45	1.288–1.638	0.001

CI = confidence interval, OR = odds ratio, Ref = reference group.

**Figure 2:**
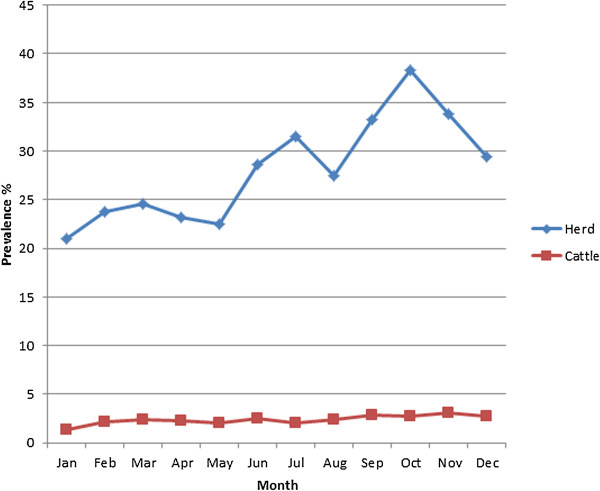
Monthly herd-level and cattle seroprevalence of bovine brucellosis in Peninsular Malaysia from 2000 to 2008.

The range of prevalence within the seropositive herds was 0.9 to 100% from an average herd size of 41 cattle. The annual mean seroprevalence for the study period was 21.7% with the highest in 2007 (26.9% CI, 24.44–29.36) and the lowest in 2000 and 2001 (18.6% CI, 16.44–20.76) (Figure 1). Table 1 shows the bovine brucellosis seroprevalence within each state. The herd-level seroprevalence rates among the states were significantly different (χ2 = 541.038, df = 10, *p* = 0.001). The highest herd level seroprevalence was observed in Pahang at 45.4% (95% CI, 35.75–51.85) and the lowest in Melaka at 10.7% (95% CI, 8.89–12.71). The herd-level seroprevalence rates varied between months within the study years, showing a significant increasing trend within the year. The months of September, October and November had the highest rates with corresponding seroprevalences of 33.2, 38.4 and 33.9%, respectively. The differences between the months were significant (χ2 = 51.596, df = 11 *p* = 0.001) (Figure 2).

Herd size was associated with the herd-level seroprevalence (χ^2^ = 1206.077, df = 2, *p* = 0.001) whereby larger herds had a higher likelihood of being seropositive. Table 1 shows the detailed descriptive and univariate analysis of herd-level data. Breeds were also associated with increased seropositivity (χ2 = 37.429, df = 1, *p* = 0.001) and beef cattle appeared to be at higher risk for seroreaction. Brahman, Bali, Kedah Kelantan and Nellore cattle had a higher likelihood of seropositivity while dairy breeds such as Friesien-Sahiwal and LID had significantly lower likelihoods (Table 1).

### Spatial distribution

**Figure 3:**
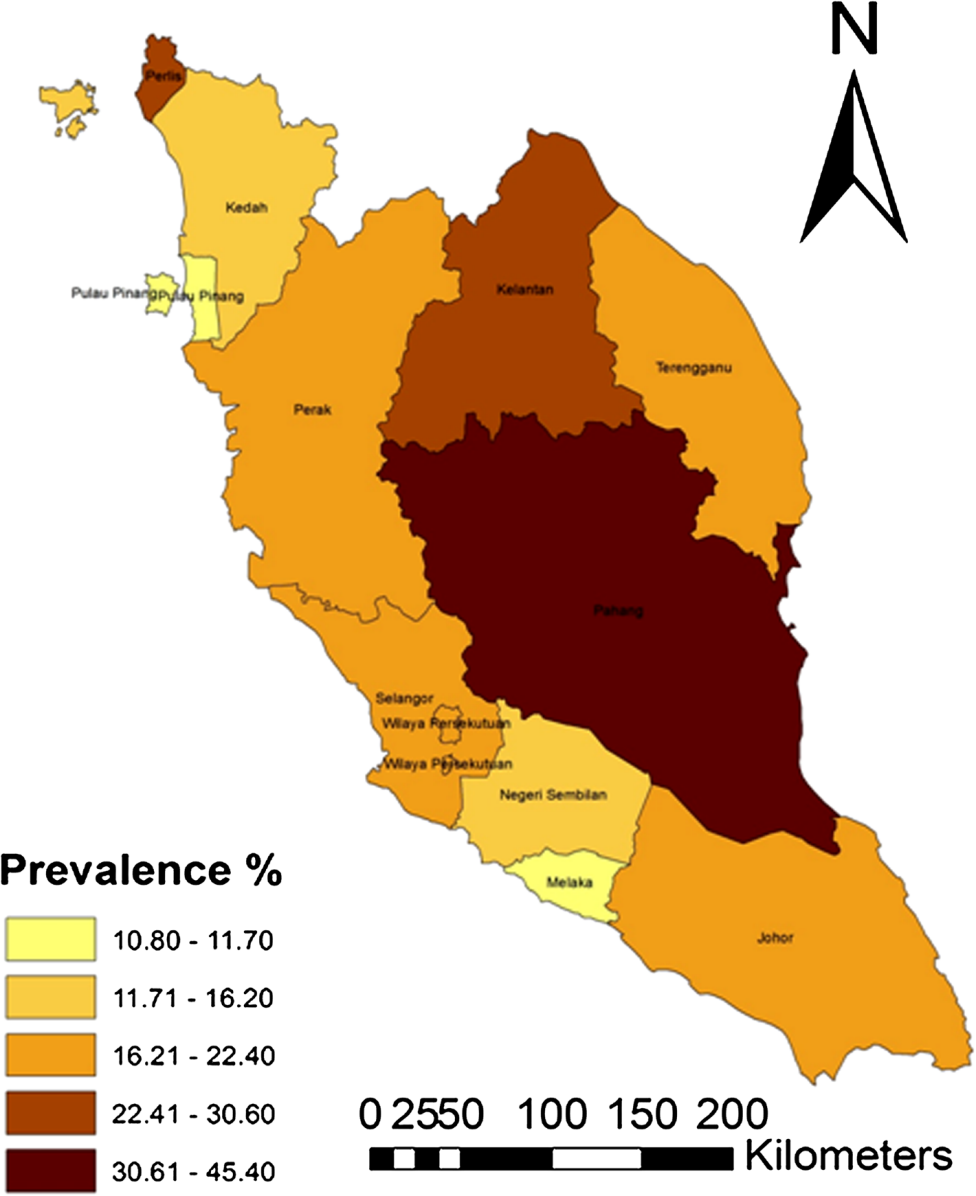
Choropleth map showing the overall prevalence of seropositive herd in Peninsular Malaysia between 2000 and 2008.

**Figure 4:**
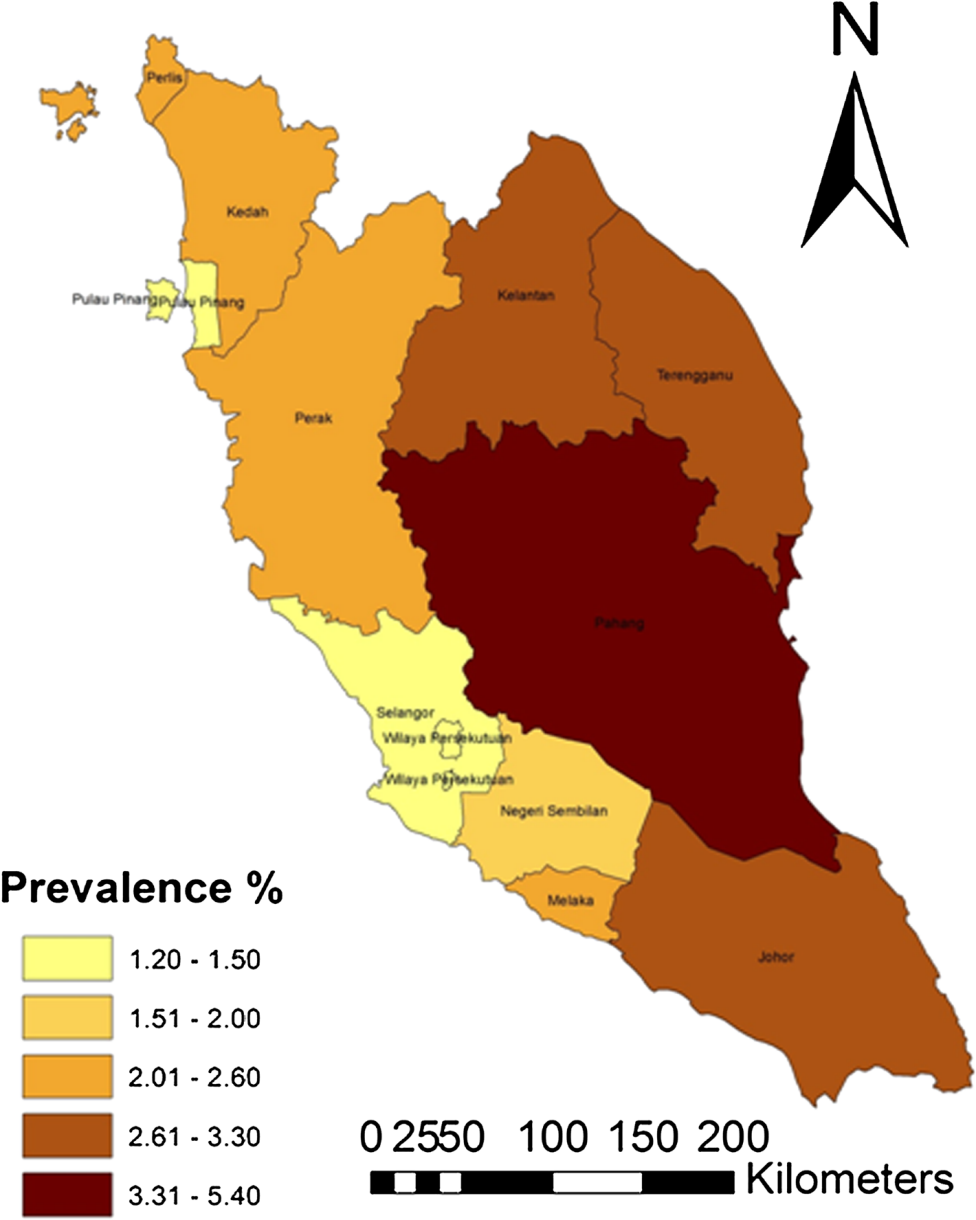
Choropleth map showing the overall prevalence of seropositive cattle in Peninsular Malaysia between 2000 and 2008.

**Figure 5:**
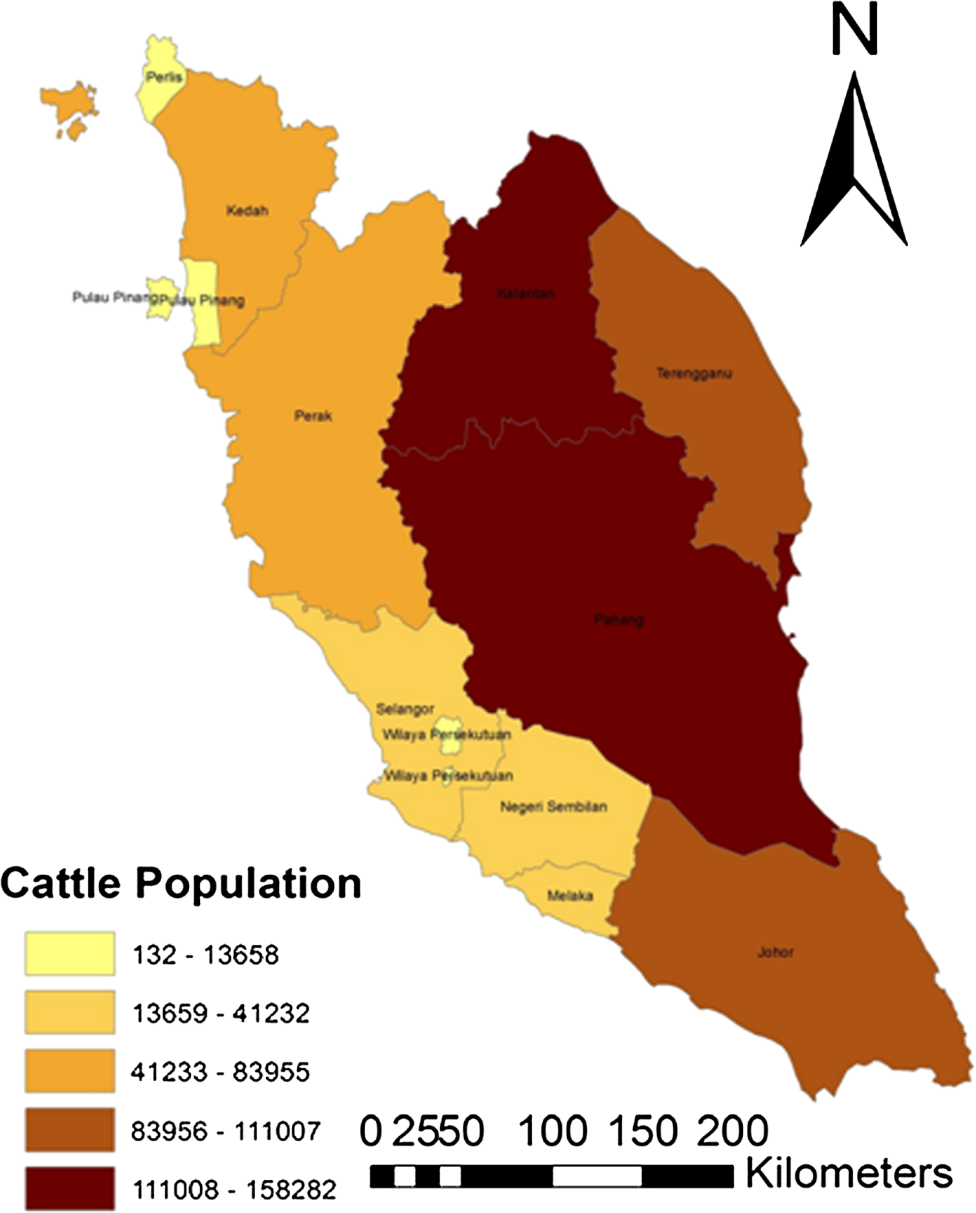
Choropleth map showing the cattle population of Peninsular Malaysia in 2008.

The choropleth map (Figures 3, 4 and 5) shows the spatial distribution of bovine brucellosis based on year and cattle population size. It appears that the sero-reactor herds tend to cluster around the central region of the peninsula with pockets of disease in the northern part of the peninsula.

## Discussion

The herd-level brucellosis seroprevalence among the cattle population of Malaysia of 21.8% (95% CI, 21.01–22.59) is slightly lower than in neighbouring countries such as Indonesia and Thailand, with rates of 27.4 and 24.1%, respectively [19,20] and other countries in the world where the disease is endemic such as Brazil, Ethiopia and Jordan, which reported herd-level seroprevalence rates of 32.4, 26.1 and 23%, respectively [21-23]. The cattle-level seroprevalence of 2.5% (95% CI; 2.45–2.55) is also lower when compared to countries where the disease is endemic such as Thailand (3.3%), Egypt (11%), Brazil (3.2%) and Nigeria (19.7%) [20,22,24,25]. The observed disparity could be attributed to various factors that were not measured in this study but which we believe could be the result of different testing and protocols used for surveillance activities, the type of cattle-rearing management system, and the level of stringency in disease-control measures in the country. In our study, the overall within-herd seroprevalence of 2.5% is a marked reduction from 21% that was previously reported in a limited geographic and sample size study [18]. In addition, another study demonstrated the success achieved through a local eradication programme, whereby the national reactor rate declined from 8.7% in 1980 to 0.4% in 1993 [13].

Herd-level seroprevalence varied significantly over the 9 years of study and showed a gentle rising trend from 2000 to 2008. We believe that the pattern observed was a function of the serosurveillance and culling activities within the affected herd. The testing and culling may have detected most of the seropositive cattle, but may have missed a small percentage, which subsequently propagated the infection to other animals and herds. Consistent with our beliefs, the cattle-level seroprevalence showed a dissimilar trend than the herd. There was a significant (3.8 to 1.1%) reduction in the reactors among cattle from 2000 to 2004, but thereafter the rate increased significantly (1.1 to 4.2%). We believe that this trend was a result of variations in the concentration of time and resources by the authorities, depending on the animal disease situation in the country. For example, the decrease in sero-reactor animals from 2000 onwards was possibly due to the intensification in controlling zoonotic diseases in the wake of the novel Nipah virus outbreak in 1998–1999 [26]. Unfortunately, from around 2004 until 2007, Malaysia suffered a few outbreaks of highly pathogenic avian influenza (HPAI), which forced the authorities to concentrate more resources, time and efforts on preventing the outbreaks from spreading and regaining a state of freedom from HPAI [27]. At the same time, Malaysia was actively involved in the Myanmar-Thailand-Malaysia (MTM) FMD eradication efforts, which may have further diverted limited resources. Consequently, surveillance activities were delayed for other diseases, including brucellosis, which thereafter led to an increased number of infected animals.

The proportion of sero-reactor herds remained relatively high (26.8%) in the period of study. This phenomenon reflects the difficulty in achieving complete disease-free status for herds that were infected. It also strengthened the argument that perhaps a low percentage of reactor animals were missed during the surveillance or culling activities, which subsequently served as the source of propagation of the disease to other susceptible animals and herds.

The observed pattern of brucellosis reactors among the states could be ascribed to multiple factors. We suspect that the choice of farm management system may be the major reason for the differences; for example, integrated farming systems (integration of cattle and crops) are highly prevalent in Pahang and part of Johor and Kelantan [28]. In this type of system, animals belonging to various owners are raised extensively on the same plantation. The nature of the system makes herd health challenging and regular veterinary services inaccessible. However, as farm management information was not included in the surveillance information, we cannot arrive at a more definitive conclusion. We also believe that there may be variations in the vigorousness of the enforcement/culling of reactor animals among states due to various reasons including available resources, time, logistics, technical help and budgetary allocations [29]. Previous studies have reported that location, region or area has a significant correlation with brucellosis seropositivity, which, according to the authors, is attributable to management practices and other agro-ecological factors [3]. Moreover, the spatial distribution of bovine brucellosis further supports the claim/point that districts in the central part of the peninsula have higher herd-level and cattle-level seroprevalence compared to other parts. The spatial mapping of the sero-surveillance data in our study also uncovered several pockets of high seroprevalence existing within a few states with relatively lower herd and cattle-level seroreactors.

Our analysis of several putative herd-level factors associated with brucellosis from the surveillance data found that herd size, year of sampling, state and month of sampling were associated with herd-level seroprevalence of bovine brucellosis in Peninsular Malaysia. Larger herds, in comparison to smaller ones, have a higher likelihood of seropositive cattle. The association of seropositivity with herd size is consistent with the results of other studies demonstrating this relationship [30,31]. Even though we do not have information on the stocking density of herd sampled in this study, we believe that there is a direct correlation between herd size and stocking density. An increase in herd size is accompanied by increased contact between animals, thus leading to cross infection [5]. This factor has been established as one of the important determinants of brucellosis seropositivity, especially given the occurrence of abortion or calving [3,32].

Among the breeds, beef cattle appear to be at a higher risk. Brahman, Bali, Kedah Kelantan and Nellore cattle had a higher likelihood of being seropositive, while the dairy breeds, such as Friesien-Sahiwal and LID, had a significantly lower likelihood. We hypothesised that the major reason for this difference was the management system of the farm, because a large proportion of beef cattle in Peninsular Malaysia are raised in extensive systems, including the integrated farming system [17]. Extensive cattle management has been consistently reported by other authors to be an important risk factor for *Brucella* seroprevalence [33,34]. It is also possible that the difference was due to other confounding variables unaccounted for in this study.

The association of month of sampling with seropositivity to *Brucella* infection is consistent with the findings of another study that reported rainfall or season to play an important role in the epidemiology of the disease [35]. In most parts of the peninsula, significantly higher rainfall occurs during the northeast monsoon from September until January (http://www.met.gov.my) and, accordingly, an increased likelihood of *Brucella* seropositivity was detected in the months of October to September in this study. Seasonal changes in the epidemiology of infectious diseases are common phenomenon in both temperate and tropical climates [36]. However, the mechanism of the change is poorly understood [36,37] and has been linked to the interaction of several intrinsic and extrinsic factors [36,38]. In the epidemiology of *B. melitensis* infection, seasonal factors have been reported to be associated with human brucellosis which, in most cases, coincide with the period of parturition among farm animals and, hence, increased exposure to farmers when attending to animals and consuming their milk [39]. In this study, we believe that extrinsic factors, such as rainfall and humidity may have contributed to the occurrence of the disease around this period, in combination with other environmental factors [38]. In addition, breeding of livestock and milk production are associated with the rainy season. Lending support to our observation, in cattle, 70% of births occur during rainfall [40]. This is accompanied by intensive shedding of *Brucella* organisms among infected animals with consequences of environmental contamination.

## Conclusions

Our findings highlight the epidemiological features of bovine brucellosis via examination of serological evidence for the presence of the organism among cattle. Bovine brucellosis was widespread within Peninsular Malaysia where a possible cluster occurred in the central region of the peninsula where integrated farming systems were commonly practiced. The herd-level seroprevalence varied but remained high within the 9 years of study, while the cattle-level seropositive rates were comparatively low, but had a more subtle trend over the study period. We believe that the two patterns reflect the difficulty in achieving a herd free from brucellosis once infected; therefore, it is worth examining the mechanism of culling *Brucella* seroreactors as currently practiced to ensure a more efficient culling system. The high herd-level seroprevalence may impact the animal industry significantly as suggested in a limited study in Pahang, where the total cost associated with bovine brucellosis was RM 3.5 million, while the cost of potential loss to the beef industry was RM 21 million [41].

The decreasing rate of cattle sero-reactors from 2000 to 2004 was possibly due to a step-up and intensification of zoonotic disease surveillance activities by authorities following the Nipah disease outbreak in the late 1990s. However, the rate increased after 2004 until 2007, possibly due to a shift in resources and time allocations from this surveillance to the control of other pertinent diseases, depending on the global and local disease situations at the time. As with other studies that use disease serosurveillance data, our study was limited by the quality of the data available, including incomplete data from individual animals sampled and inconsistent formats of data recording resulting in inconsistent information. Notwithstanding our confidence in the results as they pertain to bovine brucellosis in Peninsular Malaysia, extensive inferences from the findings should only be made with knowledge about data deficiencies.

## Methods

### Study area

Malaysia (4.1936° N, 103.7249° E) is located in Southeast Asia and comprises East Malaysia (Peninsular Malaysia) and West Malaysia (Sabah and Sarawak on Borneo Island). The two regions are separated by the South China Sea [42]. Peninsular Malaysia is comprised of 11 states and two federal territories and covers an area of 131,598 square kilometres bordering Thailand in the north and Singapore in the south. Peninsular Malaysia has an average rainfall of 2,400 mm and experiences hot and humid weather throughout the year with two monsoon seasons; the north-east monsoon from November to March and the south-east monsoon from May to September (http://www.met.gov.my). Malaysia has a relatively small cattle population size and within the years of study (2000–2008), the cattle population size ranged from 731,484 to 787,871 [43].

### Data sources

Brucellosis serosurveillance activities were performed regularly by the state veterinary departments in Malaysia, as described in the Protokol Veterinar Malaysia Penyakit *Brucella*[29]. The program allows for serological screening of cows aged four months and above twice a year. Once confirmed, all seroreactors must be culled in the government abattoir. Slaughter under the supervision of the veterinary officer is required to ensure compensation of culled cattle.

The livestock sampling and serological testing for the serosurveilance programme was performed via the state’s veterinary departments and its regional veterinary laboratories that are located throughout the peninsula. Accordingly, serum samples from cattle were tested for evidence of *Brucella* antibodies using the Rose Bengal Plate Test (RBPT) and the Complement Fixation Test (CFT), using the protocols and guidelines described by the OIE [44]. The confirmatory diagnosis for *Brucella* antibodies using the CFT was performed at the Veterinary Research Institute (VRI), Ipoh. The study was approved to be conducted by the Department of Veterinary Services, Putrajaya Malaysia.

We obtained data generated from the serological testing for bovine brucellosis from the Epidemiology and Surveillance Unit at the Department of Veterinary Services (DVS), Putrajaya, and the database at VRI, Ipoh, for years 2000 to 2008. These serosurveillance data have not been extensively analysed in the past. The data were compared and collated to improve their integrity. The data were thoroughly checked for accuracy in entry, coding and typing errors, and repeated entry of a farm in the course of one year was eliminated to ensure that a herd or farm was not overrepresented in a given year of study. The information obtained from the data includes farm names and addresses, date of sampling, location and state, breed, age range, number of animals tested and the number of animals within the tested herd.

### Data analysis

The data were managed and stored in a Microsoft Excel® (Microsoft Corporation) spreadsheet, and frequency tables were used to calculate prevalence based on state, year, herd, animal and breed. Seroprevalence rates over the 9 years were determined as the number of seropositive cattle divided by the total number of cattle sampled and confidence intervals were calculated at a 95% level. The differences between/among proportions were tested using Chi-square and univariate logistic regression statistics. Herd size was categorised as < 20, 20–40 and > 40. Age of animals on the farm was recorded as the range of the sampled animal’s age within the herd and therefore cannot be further analysed to arrive at meaningful conclusions. All statistical analyses were performed using SPSS (version 16, Chicago, IL) at a significance level of α = 0.05.

### Spatial distribution

A choropleth map was developed for herd- and cattle-level seroprevalence of bovine brucellosis from 2000 to 2008 using the software Arc GIS v9.3 (ESRI, 2006). The results of the seropositive animals and herd were aggregated into an area (state) for the spatial analysis due to the lack of exact farm/herd coordinates and to maintain confidentiality of the farms. Additional datasets on the coordinates of the states and map of Malaysia were obtained from GIS data at the Department of Survey and Mapping Malaysia (JUPEM).

## Abbreviations

HPAI: Highly pathogenic avian influenza; FMD: Foot and mouth disease; LID: Local Indian dairy; RBPT: Rose bengal plate test; CFT: Compliment fixation test; OIE: World Organization for Animal Health; VRI: Veterinary research institute; DVS: Department of Veterinary Service; JUPEM: Department of Survey and Mapping; CI: Confidence interval; df: Degree of freedom; Sd: Standard deviation; MTM: Malaysia Thailand Myanmar.

## Competing interests

The authors declare that they have no competing interests.

## Authors’ contributions

MSA carried out the study, analysed the data and drafted the manuscript. LH conceived of the study, participated in its design, coordination and helped to draft the manuscript and approved the final draft. SKB participated in the design of the study and proof reading of the manuscript. RBM helped in acquiring the data, design of the study and proof reading of the manuscript. MAZ participated in the study design and proof reading of the manuscript. AA helped in acquiring the data, design of the study and proof reading of the manuscript. All authors read and approved the final manuscript.
